# “High-Risk Breast Cancer Screening in BRCA1/2 Carriers Leads to Early Detection and Improved Survival After a Breast Cancer Diagnosis”

**DOI:** 10.3389/fonc.2021.683656

**Published:** 2021-09-02

**Authors:** Shay Shraga, Albert Grinshpun, Aviad Zick, Luna Kadouri, Yogev Cohen, Ofra Maimon, Yael Adler-Levy, Galina Zeltzer, Avital Granit, Bella Maly, Einat Carmon, Vardiella Meiner, Tamar Sella, Tamar Hamburger, Tamar Peretz

**Affiliations:** ^1^Sharett Institute of Oncology, Hadassah-Hebrew University Medical Center, Jerusalem, Israel; ^2^Faculty of Medicine, The Hebrew University of Jerusalem, Jerusalem, Israel; ^3^Radiology Department, Hadassah-Hebrew University Medical Center, Jerusalem, Israel; ^4^Pathology Department, Hadassah-Hebrew University Medical Center, Jerusalem, Israel; ^5^Surgery Department, Hadassah-Hebrew University Medical Center, Jerusalem, Israel; ^6^Department of Genetic and Metabolic Diseases, Hadassah-Hebrew University Medical Center, Jerusalem, Israel

**Keywords:** breast cancer, *BRCA1/2*, high-risk, survival, screening, downstaging

## Abstract

**Background:**

Germline *BRCA1/2* pathogenic variant (PV) carriers have high lifetime risk of developing breast cancer and therefore subjected to intense lifetime screening. However, solid data on the effectiveness of high-risk screening of the *BRCA1/2* carrier population is limited.

**Patients and Methods:**

Retrospectively, we analyzed 346 women diagnosed with breast tumors. Patients were divided according to the timing of *BRCA1/2* PVrecognition, before (BRCA-preDx awareness, N = 62) or after (BRCA-postDx awareness group, N = 284) cancer diagnosis.

**Results:**

Median follow-up times were 131.42 and 93.77 months in the BRCA-preDx awareness and BRCA-postDx awareness groups, respectively. In the BRCA-preDx awareness group, 78.7% of the patients had invasive tumors and 21.3% were diagnosed with pure ductal carcinoma *in situ*. In contrast, in the BRCA-postDx awareness group over 93% of women were diagnosed with invasive cancer and only 6.4% had *in situ* disease. The mode of tumor detection differed significantly between the groups: 71.9% in the BRCA-postDx awareness group and 26.2% in the BRCA-preDx awareness group were diagnosed after personally palpating a lump. Tumor size and nodal involvement were significantly more favorable in the BRCA-preDx awareness group. T stage was significantly lower in the BRCA-preDx awareness group: 54.84% at T1 and 20.96% at Tis. In the BRCA-postDx awareness group, only 37.54% were at T1 and 6.49% at Tis. The N stage was also significantly lower in the BRCA-preDx awareness group: 71% had no lymph node metastases, compared with 56.1% in the BRCA-postDx awareness group. Additionally, therapeutic procedures varied between the groups: BRCA-preDx awareness group patients underwent more breast conserving surgeries. Axillary lymph node dissection was done in 38% of women in the BRCA-postDx awareness group and in only 8.7% of the BRCA-preDx awareness group patients. Interestingly, improved survival was found among patients who underwent high-risk screening (hazard ratio=0.34).

**Conclusions:**

High-risk screening might facilitate downstaging of detected breast tumor among *BRCA1/2* carrier population.

## Introduction

Breast cancer is the most prevalent non-cutaneous cancer among women (1). In general, once diagnosed, early and accurate detection of the tumor size and degree of spreading is very important, since treatment in the early stages of the disease can improve the prognosis and save lives (2).

Women who carry a *BRCA1* or *BRCA2* pathogenic variant (PV) are at an increased risk of developing breast cancer. These women hold a lifetime risk as high as 60% to 90% (3), and also a risk of developing it at younger age than women in the general population (4). Finding *BRCA1/2* PV significantly alters medical management (5) and prompts earlier and more frequent screening and risk-reduction surgeries (6).

As part of the high-risk screening, the National Comprehensive Cancer Network’s (NCCN) Guidelines state that for *BRCA1/2* carriers, annual magnetic resonance imaging (MRI) and clinical breast exams should start at age 25, and mammograms should start at the age of 30 (7).

There is evidence that for carriers of *BRCA2* PV, a combination of MRI screening and annual mammography can have a survival benefit (8, 9). On the other hand, for *BRCA1* PV carriers the high-risk screening appears to be less effective. This might be because of the high prevalence of triple-negative breast cancer (TNBC) in *BRCA1* carriers, an aggressive subtype with a poor prognosis (10).

Mammography can detect lesions at a minimal size of 1 mm and reveal breast cancer several years before it can be detected in a physical examination (11). MRI is even more sensitive screening modality than mammography alone (11), and the combination of the two is the most sensitive method for detecting breast cancer (12). However, information on the effectiveness of high-risk screening for the *BRCA* carrier population is limited. Frequent physical examination, mammography, and MRI, starting as early as possible in the high-risk population of *BRCA* carriers, are commonly used.

Here we aimed to determine whether high-risk screening has the potential to benefit *BRCA1/2* PV carrier population.

## Methods

### Study Design & Patients

This retrospective study included 346 high-risk women who were diagnosed with breast cancer in 1996–2020 at the Oncology Department of the Hadassah-Hebrew University Medical Center in Jerusalem. The study focused on patients diagnosed during 1996-2020 due to the data accessibility.

The high-risk women are *BRCA1/2* PV carriers. The 346 patients were divided into two groups in order to determine the impact of high-risk screening. The BRCA-preDx awareness group comprised 62 women who knew that they were carriers of *BRCA* PVs. Therefore, they were offered intensified screening prior to breast cancer diagnosis. The BRCA-postDx awareness group consisted of 284 patients who first were diagnosed with breast cancer and only then they were found to carry *BRCA* PV. Therefore, the BRCA-postDx awareness group was not under high-risk screening. In 2009, the Israeli Ministry of Health added the reimbursement of annual MRI as a standard screening modality for *BRCA1/2* carriers. Therefore, we re-analyzed the data based on a cutoff at 2009 and separated the patients diagnosed before and after 2009 in each group.

Before 2009, the recommended screening included biannual clinical evaluation, breast ultrasound from the age of 25 years or 10 years prior to the age of diagnosis of family member, whatever comes first, and annual mammography from the age of 35 years. After 2009, annual MRI as a standard screening modality for *BRCA1/2* carriers.

The Hadassah Institutional Review Board approved the study and all patients gave written informed consent.

Clinical data were obtained from electronic medical records of Hadassah Medical Center. The data included demographics and information regarding the breast cancer: tumor size, lymph node status and distant metastasis (TNM), date of first diagnosis, pathology, receptor status, type of surgery, *BRCA* PV type, how the first diagnosis was made, family history, and follow-up.

Exclusion criteria were prior diagnosis of cancer and high-risk mutation other than *BRCA* PV.

### Statistical Analysis

Association between two categorical variables was tested using the χ2 test and Fisher’s exact test. Continuous variables were compared between two independent groups by use of the two-sample t-test or the non-parametric Mann-Whitney test. The non-parametric test was used for variables that were not normally distributed. The Kaplan-Meier survival model was used for assessing survival, with the log-rank test for the comparison of survival curves. The Cox regression model was applied as the multivariable model for survival. Lead time bias correction was done as described previously (13), it assumes an exponential distribution of the sojourn time, the period during which the tumor is asymptomatic but screen-detectable, with a rate of transition to symptomatic disease λ. Thus, 1/λ is the mean sojourn time and is typically around 4. Duffy calculates an expected additional follow-up time to be subtracted from the calculated time-to-event of the study group. Where T is the last know follow-up time: follow-up correction time = (1-e^(-λT))/λ.

All statistical tests used were two-tailed, and a *P*-value of 0.05 or less was considered statistically significant. We used SSPS software for the statistical analysis.

## Results

### Study Population

The total study population included 346 female patients (**Table 1**). The median age at diagnosis was 45.9 years (range 25–81). The BRCA-preDx awareness group included 62 patients and the BRCA-postDx awareness group 284 with similar mean age at diagnosis. In the BRCA-preDx awareness group, the majority of the patients (55/62, 88.7%) had at least one immediate family member who had a history of cancer, and all had a family history of cancer. In the BRCA-postDx awareness group, only 64.2% (177/276) of the patients had at least one immediate family member who had history of cancer, and 12% (33/276) had no family history of cancer (*P* < 0.001).

**Table 1:** Characteristics of the study population.

Characteristic	BRCA-pre Dx awareness (*N*= 62)	BRCA-post Dx awareness (*N*=284)	*P* value	All (*N* = 346)
**Age mean (SD), yr**	47.4 (12.3)	45.6 (11.65)	**0.27**	45.9 (11.8)
**Age<50**	35/61 (57.4)	179/277 (64.6)		214/338 (63.3)
**Age >50**	26/61 (42.6)	98/277 (35.4)		124/338 (36.7)
**Female sex, no (%)**	62 (100)	284 (100)		346 (100)
**Months of follow-up, median (min, max)**	131.42 (3.06,271.9)	93.77 (0.95,282.4)		99.8 (0.95,282.35)
**Ancestry, no (%)**				
Ashkenazi Jewish	53/62 (85.5)	228/281^a^ (81.1)	**0.4**	281/343 (81.9)
Sephardi Jewish	8/62 (12.9)	36/281^a^ (12.8)		44/343 (12.8)
Other/Unknown	1/62 (1.6)	17/281^a^ (6)		18/343 (5.2)
**Family history, no (%)**			**<0.001**	
First degree	55/62 (88.7)	177/276^b^ (64.2)		232/338 (68.6)
Second degree	5/62 (8.1)	66/276^b^ (23.9)		71/338 (21)
Third degree	2/62 (3.2)	0/276^b^ (0)		2/338 (0.6)
None	0/62	33/276^b^ (12)		33/338 (9.8)

a Missing data of ancestry was missing for three patients.

b Missing data of family history was missing for eight patients.

(Right) All patients. (Left) Comparison of the BRCA-preDx awareness group with the BRCA-postDx awareness group.

The patients’ breast tumors characteristics are described in detail in **Table 2**. Interestingly, *BRCA1* PV was more frequent in the BRCA-preDx awareness group (48/62, 77.4%) compared with the BRCA-postDx awareness group (170/284, 59.9%; *P* = 0.009). One patient was positive for both *BRCA1/2* PVs. DCIS (ductal carcinoma *in situ*) was a more common pathology result in the BRCA-preDx awareness group, with 21.3% (13/61) of the patients having a pure DCIS at the time of diagnosis, compared with the BRCA-postDx awareness group’s 6.4% (18/280; *P* = 0.001). IDC (invasive ductal carcinoma) was the pathologic diagnosis in 78.7% (48/61) of the patients in the BRCA-preDx awareness group, and in 90.4% (253/280) of the patients in the BRCA-postDx awareness group (*P* = 0.001). There were no statistically significant differences in receptor status or tumor grade between the groups.

**Table 2:** Breast tumor characteristics.

Characteristic	BRCA-pre Dx awareness (*N*= 62)	BRCA-post Dx awareness (*N*=284)	*P* value
***BRCA1*** positive	48/62 (77.4)	170/284 (59.9)	0.009
***BRCA2*** positive	15/62 (24.2)	114/284 (40.1)	0.019
**Receptor status**			0.248
**Invasive**			
ER/PR positive, HER2 negative	13/60^a^ (21.67)	103/277^b^ (37.2)	
ER/PR negative, HER2 positive	6/60^a^ (10)	15/277^b^ (5.4)	
Triple negative	25/60^a^ (41.67)	100/277^b^ (36.1)	
ER/PR positive, HER2 positive	4/60^a^ (6.67)	27/277^b^ (9.74)	
ER/PR positive, HER2 NA	0 (0)	6/277^b^ (2.2)	
ER/PR negative, HER2 NA	0 (0)	9/277^b^ (3.24)	
***In situ***			
ER/PR positive	9/60^a^ (15)	15/277^b^ (5.42)	
ER/PR negative	3/60^a^ (5)	2/277^b^ (0.72)	
**Grade**			0.119
**Invasive**			
Grade 1	3/46^c^ (6.5)	9/232^d^ (3.9)	
Grade 2	8/46^c^ (17.4)	59/232^d^ (25.4)	
Grade 3	28/46^c^ (60.9)	150/232^d^ (64.7)	
***In situ***			
Low	2/46^c^ (4.3)	3/232^d^ (1.3)	
Intermediate	2/46^c^ (4.3)	6/232^d^ (2.6)	
High	3/46^c^ (6.5)	5/232^d^ (2.2)	
**Invasive vs. not invasive pathology**	** **	** **	0.001
**DCIS**			
Positive	13/61^e^ (21.3)	18/280^f^ (6.4)	
Negative	48/61^e^ (78.7)	262/280^f^ (93.6)	
**IDC**			
Positive	48/61^e^ (78.7)	253/280^f^ (90.4)	
Negative	13/61^e^ (21.3)	27/280^f^ (9.64)	
**ILC**			
Positive	0/61^e^	9/280^f^ (3.2)	
Negative	61/61^e^ (100)	271/280^f^ (96.8)	

a Missing data of Receptor status was missing for two patients.

b Missing data of Receptor status was missing for seven patients.

c Missing data of grade was missing for sixteen patients.

d Missing data of grade was missing for fifty two patients.

e Missing data of Pathology was missing for one patient.

f Missing data of Pathology was missing for four patients.

(Right) All patients. (Left) Comparison of the BRCA-preDx awareness group with the BRCA-postDx awareness group. ER, estrogen receptor; PR, progesterone receptor; HER2, human epidermal growth factor receptor 2; NA, not applicable; DCIS, ductal carcinoma in situ; IDC, invasive ductal carcinoma; LCIS, lobular carcinoma in situ; ILC, invasive lobular carcinoma.

### Mode of Tumor Detection

**Figure 1:**
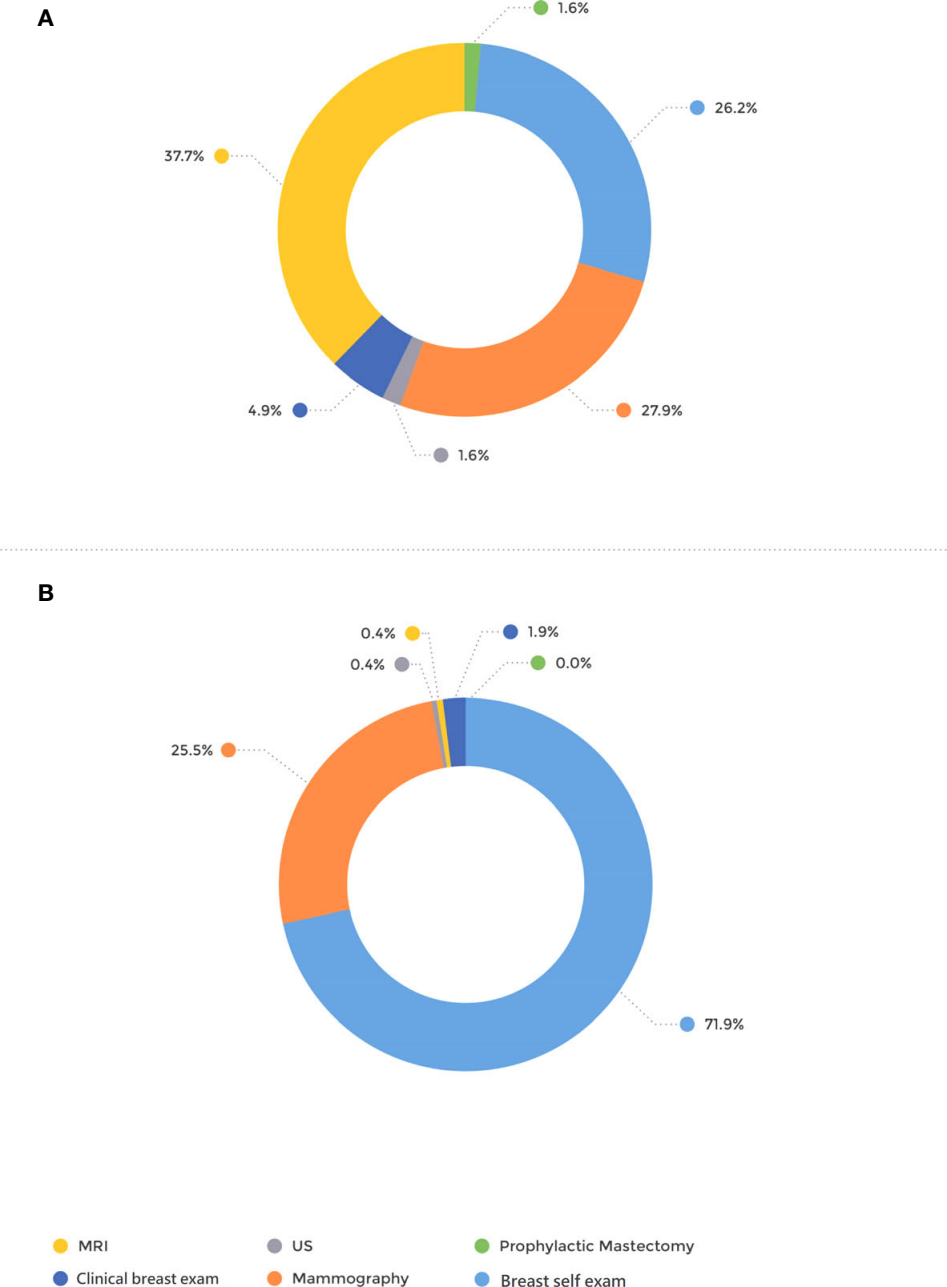
Mode of tumor detection. **(A)** BRCA-preDx awareness group. **(B)** BRCA-postDx awareness group. MRI, magnetic resonance imaging; US, ultrasound.

The mode of detection differed significantly between the groups; 71.9% of the patients in the BRCA-postDx awareness group were diagnosed with breast cancer after they personally palpated a lump in their breast (**Figure 1**). That appears in contrast to the BRCA-preDx awareness group, where 26.2% of the patients personally palpated a lump (*P* < 0.001). In the BRCA-preDx awareness group, MRI was the diagnostic tool in 37.7% (23/61) of the cases, versus the BRCA-postDx awareness group where it accounted for only one case (0.4%, 1/267). In patients of younger ages, tumors were detected by self-palpation more frequently than by mammography in both study groups (**Supplementary Figure 1**).

### Tumor Stage at Diagnosis

**Figure 2:**
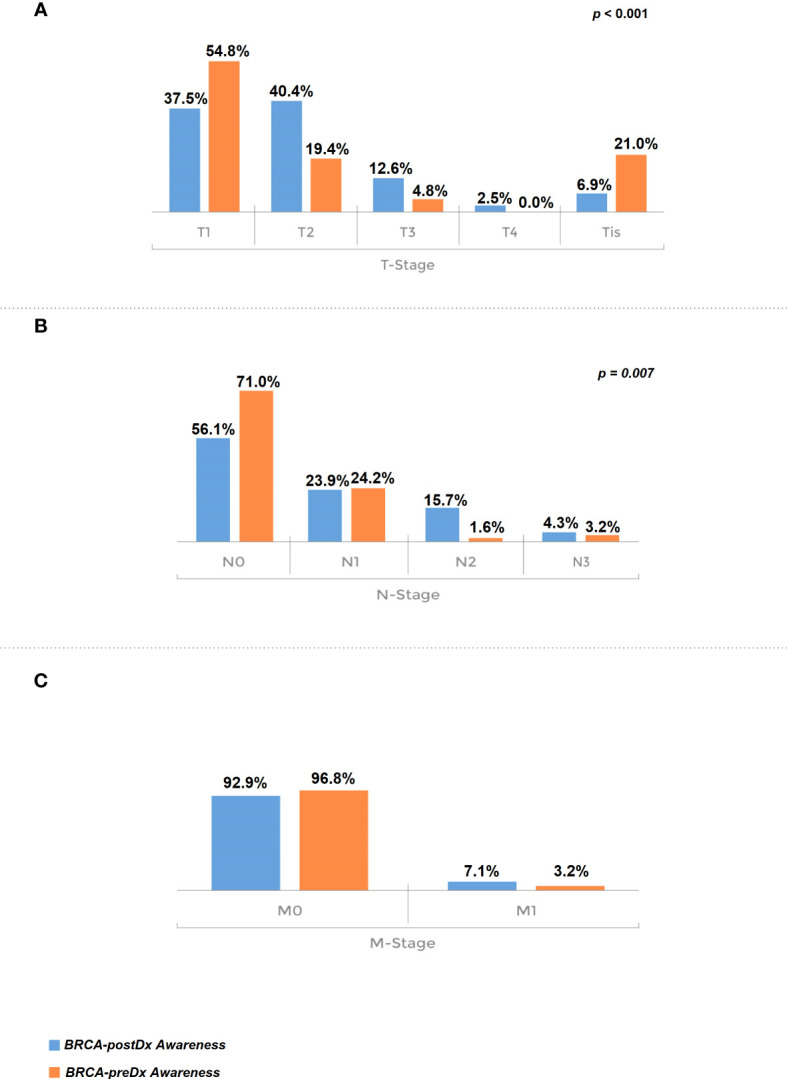
Tumor stage at diagnosis. **(A)** T stage, comparison of the BRCA-preDx awareness group with the BRCA-postDx awareness group. **(B)** N stage: comparison of the BRCA-preDx awareness group with the BRCA-postDx awareness group. **(C)** M stage: comparison of the BRCA-preDx awareness group with the BRCA-postDx awareness group.

Furthermore, TNM staging was significantly more favorable in the BRCA-preDx awareness group. T stage was significantly lower in the BRCA-preDx awareness group (**Figure 2**): the majority of the patients were diagnosed at T1 (34/62, 54.8%), and 21% (13/62) of the patients were diagnosed at Tis. In the BRCA-postDx awareness group, only 37.5% (104/277) of the patients were diagnosed at T1 and 6.9% (19/277) of patients at Tis (*P* < 0.001). The N stage was also significantly lower in the BRCA-preDx awareness group. Within the BRCA-preDx awareness group, 71% of the patients were diagnosed with no lymph nodal metastases (44/62), while in the BRCA-postDx awareness group only somewhat more than half of the patients were diagnosed with no lymph node metastases (157/280, 56.1%; *P* = 0.007). Distant metastases were found in 3.4% of the patients in the BRCA-preDx awareness group and in 7.1% of the patients in the BRCA-postDx awareness group (non-significant trend).

### Therapeutic Procedures Among the Study Population

Significantly, less patients in the BRCA-postDx awareness group underwent breast conserving surgeries compared with the BRCA-preDx awareness group (**Table 3**). Thirty-eight percent of patients from the BRCA-postDx awareness group had axillary lymph node dissection (ALND) compared with only 8.8% (5/57, *P* < 0.001) in the BRCA-preDx awareness group. In addition, mastectomy was more frequently performed within the BRCA-postDx awareness group compared with the BRCA-preDx awareness group. Prophylactic bilateral mastectomy after diagnosis was less common in the BRCA-postDx awareness group (55/280, 19.6%) than in the BRCA-preDx awareness group (29/57, 50.9%; *P* < 0.001).

**Table 3:** Therapeutic procedures among the study population.

Location	Procedure	BRCA-pre Dx awareness (*N* = 62)	BRCA-post Dx awareness (*N* = 284)	*P* value
**Breast**	Lumpectomy	38.6 (22/57)^a^	48.2 (135/280)^b^	**<0.001**
	Unilateral mastectomy	10.5 (6/57)^a^	25 (70/280)^b^	
	Bilateral mastectomy	50.9 (29/57)^a^	19.6 (55/280)^b^	
	Inoperable	3.4 (2/59)^a^	7.1 (20/280)^b^	
**Axilla**	Sentinel	36.8 (21/57)^a^	29.3 (82/280)^b^	**<0.001**
	Dissection	8.8 (5/57)^a^	38.6 (108/280)^b^	

a Missing data of Procedure was missing for five patients.

b Missing data of Procedure was missing for four patients.

(Right) All patients. (Left) Comparison of the BRCA-preDx awareness group with the BRCA-postDx awareness group. Unless otherwise indicated, data are percentages (number of patients/total with known variable).

### Outcomes Following Inclusion of MRI in the National Health Services

The BRCA-preDx awareness group included 29 patients diagnosed before and 33 patients after 2009, and the BRCA-postDx awareness group included 183 patients diagnosed before 2009 and 101 after 2009 (**Table 4**). Not surprisingly, the wider use of MRI had a clear effect. Until 2009, pathology results were similar between the groups, however, after 2009 the differences became significant: 31.3% (10/32) of patients had pure DCIS in the BRCA-preDx awareness group, while only 8.1% (8/99) did within the BRCA-postDx awareness group(*P* = 0.005). Additionally, before 2009, the tumor was diagnosed through MRI in 13.8% (4/29) of the patients in the BRCA-preDx awareness group (*P* < 0.001), and this rose to 59.4% after 2009 (*P* < 0.001). Furthermore, the staging of invasive tumors at diagnosis was also affected, and a major downstaging in the BRCA-preDx awareness group was noted (T2–T4: 34.5% before 2009, 15.2% after 2009; *P* = 0.012). The difference in N stage between BRCA-preDx awareness and BRCA-postDx awareness is apparent only after 2009.

**Table 4:** Outcomes following inclusion of MRI in the national health services.

Characteristic	Before 2009		*P* value	After 2009		*P* value
	BRCA-pre Dx awareness (*N*=29)	BRCA-post Dx awareness(*N*=183)		BRCA-pre Dx awareness (*N*=33)	BRCA-post Dx awareness (*N*=101)	
Pathology^a^						
			0.516			0.005
DCIS						
Positive	(3/29) 10.3	5.5 (10/181)		31.3 (10/32)	8.1 (8/99)	
Invasive disease						
Positive	89.7(26/29)	94.5 (171/181)		68.8 (22/32)	91.9 (91/99)	
Mode of cancer detection^b^						
			<0.001			<0.001
Breast self exam	37.9 (11/29)	73.4 (124/169)		15.6 (5/32)	69.4 (68/98)	
Mammography	34.5 (10/29)	24.9 (42/169)		21.9 (7/32)	26.5 (26/98)	
US	3.4 (1/29)	0.6 (1/169)		0 (0/32)	0 (0/98)	
MRI	13.8 (4/29)	0 (0/169)		59.4 (19/32)	1 (1/98)	
Clinical breast exam	6.9 (2/29)	1.2 (2/169)		3.1 (1/32)	3.1 (3/98)	
Prophylactic Mastectomy	3.4 (1/29)	0 (0/169)		0 (0/32)	0 (0/98)	
Procedure^c^						
			0.091			<0.001
Lumpectomy	46.4 (13/28)	48 (86/179)		31 (9/29)	48.5 (49/101)	
Mastectomy	14.3 (4/28)	29.1 (52/179)		6.9 (2/29)	17.8 (18/101)	
Bilateral mastectomy	39.3 (11/28)	19.6 (35/179)		62.1 (18/29)	19.8 (20/101)	
Inoperable	0 (0/28)	3.4 (6/179)		6.1 (2/33)	13.9 (14/101)	
Axilla			0.004			0.011
Sentinel	39.3 (11/28)	21.2 (38/179)		34.5 (10/29)	43.6 (44/101)	
Dissection	17.9 (5/28)	51.4 (92/179)		0 (0/29)	15.8 (16/101)	
Stage at diagnosis						
T stage^d^			0.079			<0.001
Tis	(3/29)10.3	5.6 (10/180)		30.3 (10/33)	9.3 (9/97)	
T1	55.2 (16/29)	40 (72/180)		54.5 (18/33)	33 (32/97)	
T2	34.5 (10/29)	37.8 (68/180)		6.1 (2/33)	45.4 (44/97)	
T3	0 (0/29)	14.4 (26/180)		9.1 (3/33)	9.3 (9/97)	
T4	0 (0/29)	2.2 (4/180)		0 (0/33)	3.1 (3/97)	
N stage^e^			0.166			0.051
N0	72.4 (21/29)	53.6 (97/181)		69.7 (23/33)	61.2 (60/98)	
N1	24.1 (7/29)	26.5 (48/181)		24.2 (8/33)	19.4 (19/98)	
N2	3.4 (1/29)	14.9 (27/181)		0 (0/33)	16.3 (16/98)	
N3	0 (0/29)	5 (9/181)		6.1 (2/33)	3.1 (3/98)	
M stage^f^			0.364			0.73
M0	100 (29/29)	94.5 (171/181)		93.9 (31/33)	90.1 (91/101)	
M1	0 (0/29)	5.5 (10/181)		6.1 (2/33)	9.9 (10/101)	

a Missing data of Pathology was missing for five patients.

b Missing data of Mode of cancer detection was missing for eighteen patients.

c Missing data of Procedure was missing for five patients.

d Missing data of T stage made was missing for seven patients.

e Missing data of N stage made was missing for five patients.

f Missing data of M stage made was missing for two patients.

(Left) Comparison of the BRCA-preDx awareness group with the BRCA-postDx awareness group before the inclusion of MRI. (Right) Comparison of the BRCA-preDx awareness group with the BRCA-postDx awareness group after the inclusion of MRI. DCIS, ductal carcinoma in situ; IDC, invasive ductal carcinoma; LCIS, lobular carcinoma in situ; ILC, invasive lobular carcinoma; MRI, magnetic resonance imaging; US, ultrasound. Unless otherwise indicated, data are percentages (number of patients/total with known variable).

### Overall Survival

**Figure 3:**
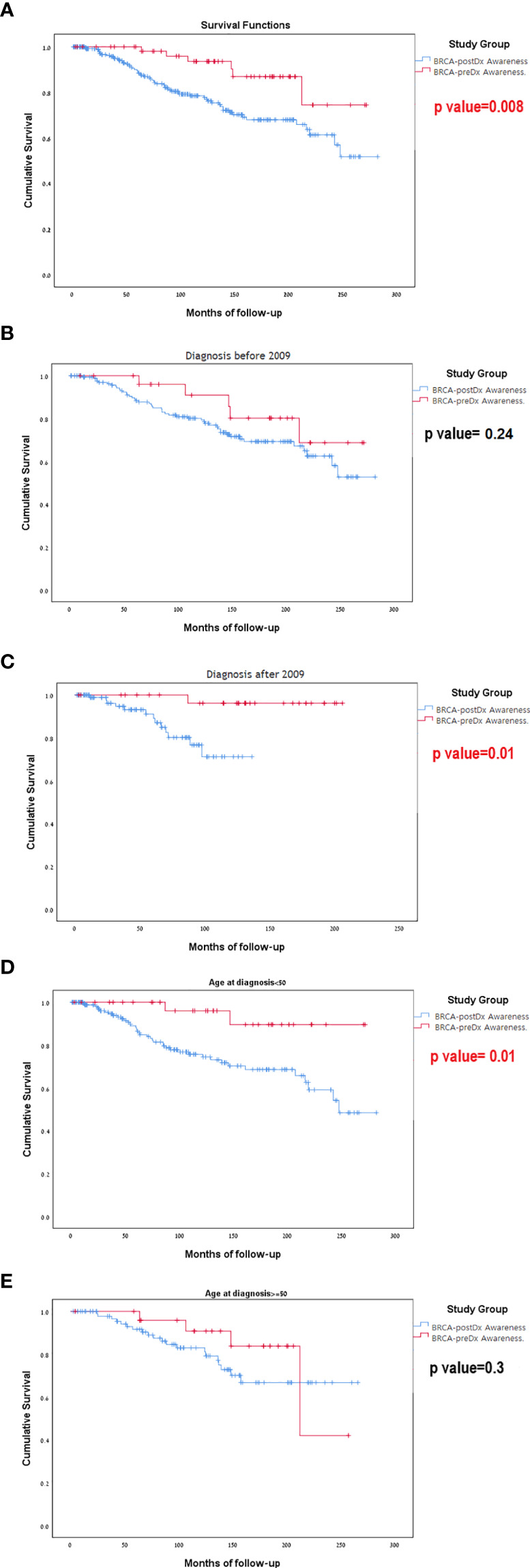
Kaplan-Meier curves for overall survival. **(A)** BRCA-preDx awareness cohort and BRCA-postDx awareness cohort. **(B)** BRCA-preDx awareness cohort and BRCA-postDx awareness cohort before 2009. **(C)** BRCA-preDx awareness cohort and BRCA-postDx awareness cohort after 2009. **(D)** BRCA-preDx awareness cohort and BRCA-postDx awareness cohort before the age of 50 years. **(E)** BRCA-preDx awareness cohort and BRCA-postDx awareness cohort after the age of 50 years.

During our study period (1996–2020), 72 patients died. Sixty-six (23.2%) women died in the 238 BRCA-postDx awareness group, compared with six patients (9.7%) from the BRCA-preDx awareness group. In the overall study period analysis, we have found significantly improved survival in the BRCA-preDx awareness group (*P* = 0.008, **Figure 3A**). Correction for lead time bias was done and the results remained statistically significant (**Supplementary Figure 2**, p=0.0135). Further univariate analysis of our data found that there is no statistical difference in survival when patients are divided according to BRCA status (**Supplementary Table 1**, **Supplementary Figure 3**). Additional factors which are also significantly associated with improved survival are PR status and TNM staging. In a multivariate analysis, only PR status and M stage were significant (**Supplementary Table 1**).

In the sub-group analysis, there was no significant different in survival for between the *BRCA-*preDX group and the *BRCA*-postDx group prior to 2009 when MRI was not covered (p=0.237, **Figure 3B**): but there was statistically significant difference in survival after 2009 when MRI introduced (p=0.011, **Figure 3C**). Young women (age<50) with a diagnosis of breast cancer and *BRCA-*preDx awareness had improved survival (p=0.01) compared to women who were identified to harbor a *BRCA* PV after their breast cancer diagnosis (**Figure 3D**). Among older women (age>50), awareness of their *BRCA* status before their diagnosis of breast cancer did not improve their survival compared to women identified to have *BRCA* after their initial diagnosis (p=0.305, **Figure 3E**).

## Discussion

In this retrospective study, we found a potential downstaging effect of high-risk screening with several key differences between patients who were offered high-risk screening and those who were not.

First, breast cancer in the BRCA-preDx awareness group was more often detected by imaging, mostly MRI, while in the BRCA-postDx awareness group self-palpation was most prevalent. We attribute this difference to the use of MRI in the BRCA-preDx awareness group, as suggested by international guidelines (7, 14) and previous studies that have shown that MRI has superior sensitivity in the *BRCA1/2* carrier population (15, 16). Second, we show that more *in situ* pathology was found in the BRCA-preDx awareness group. Moreover, invasive tumors’ TNM staging was significantly more favorable in the BRCA-preDx awareness group: the tumors were smaller, less axillary involvement and importantly, axillary surgeries were more conservative. These findings are in line with the scientific background on which the current guidelines were established (7): MRI is more sensitive than mammography for women at high risk of developing breast cancer (17–19). The use of combined screening modalities elevates the sensitivity of the examination (15, 19) and can lead to detection at favorable stages compared with mammography alone (20). Additionally, mammography alone has a higher false-negative rate in *BRCA* PVcarriers (21), in high-density breast tissue (21–23) and in rapidly growing aggressive tumors (24, 25). All of these are more frequent characteristics among high-risk young women (7). To strengthen our findings, sub-analysis using the year of the beginning of widespread use of MRI made the above-mentioned differences even more apparent (e.g., improved staging and more *in situ* tumors after 2009).

Even though studies show that the addition of MRI is superior to mammography alone in *BRCA1/2* carriers in detecting breast cancer, the currently available data has not shown a clear survival benefit from many of the above screening recommendations (16, 26–28). For example, the starting age and the age limit for high-risk screening are not well-known and based on limited observational data (26, 28). The interval time between each screening is also not well established, and there is only partial comparative information regarding different interval durations based on age (15).

A small hint of improved efficacy of high-risk screening was shown in a recent paper by Hadar et al. (29) The authors described a limited population (42 out of 105 *BRCA1/2* carriers) who knew that they were *BRCA* carriers prior to cancer diagnosis, were under high-risk screening, and had better outcomes with a possible survival advantage. Our work shows an interesting improvement in survival in young women who underwent high-risk screening. Taken together, our data imply that the introduction of MRI-based screening was the probable driver behind this survival gain due to its ability to detect smaller tumors.

Our study has several limitations. The compliance with high-risk screening in the BRCA-preDx awareness group patients is largely unknown. We do not have additional information on subsequent procedures (e.g. salpingo-oophorectomy) or therapies such as chemotherapy and hormonal therapies. Additionally, we lack data about cancer recurrence rates in both groups. However, we believe that the long follow-up period compensates for the above-mentioned drawbacks and adds important insights to those from published cohorts (16).

In summary, our data emphasize the importance of high-risk MRI-based screening among *BRCA1/2* PV carriers. This study shows a favorable effect on staging for women who have had awareness of their *BRCA* status before cancer diagnosis and had participated in intensified screening. Further studies are needed to refine the optimal screening protocols to maximize the survival benefit from high-risk screening programs.

## Data Availability Statement

The original contributions presented in the study are included in the article/**Supplementary Material**. Further inquiries can be directed to the corresponding author.

## Ethics Statement

The studies involving human participants were reviewed and approved by Hadassah Medical Center IRB. The patients/participants provided their written informed consent to participate in this study.

## Author Contributions

Conception and design: SS, ALG, TS, and TP. Collection and assembly data: SS, ALG, TH, YC, LK, OM,YA-L, GZ, AG, BM, EC, VM, TS, and TP. Data analysis and interpretation: SS, ALG, TH, YC, and TP. Manuscript writing: SS, ALG, AZ, and TP. SS and ALG had equally contributed to this manuscript. All authors contributed to the article and approved the submitted version.

## Funding

ALG was supported by the 2020 Conquer Cancer-ICRF Career Development Award.

## Conflict of Interest

The authors declare that the research was conducted in the absence of any commercial or financial relationships that could be construed as a potential conflict of interest.

## Publisher’s Note

All claims expressed in this article are solely those of the authors and do not necessarily represent those of their affiliated organizations, or those of the publisher, the editors and the reviewers. Any product that may be evaluated in this article, or claim that may be made by its manufacturer, is not guaranteed or endorsed by the publisher.

## Supplementary Material

Supplementary Figure 1 How the first diagnosis was made with regard to age. BRCA-preDx awareness group (right) and BRCA-postDx awareness group (left). MRI, magnetic resonance imaging; US, ultrasound.

Supplementary Figure 2 Survival analysis following lead-time bias correction. BRCA-preDx awareness cohort and BRCA-postDx awareness cohort.

Supplementary Figure 3 Survival analysis according to *BRCA1* and *BRCA2* status. BRCA-preDx and BRCA-postDx awareness cohort separated according to *BRCA1/2*.

Supplementary Table 1 Univariate and multivariate analyses of overall survival.
