# Supplementary figures and images for: “High-Risk Breast Cancer Screening in BRCA1/2 Carriers Leads to Early Detection and Improved Survival After a Breast Cancer Diagnosis”
Source: Front Oncol. 2021 Sep 2;11:683656. doi: 10.3389/fonc.2021.683656 (PMC8443779; doi:10.3389/fonc.2021.683656)

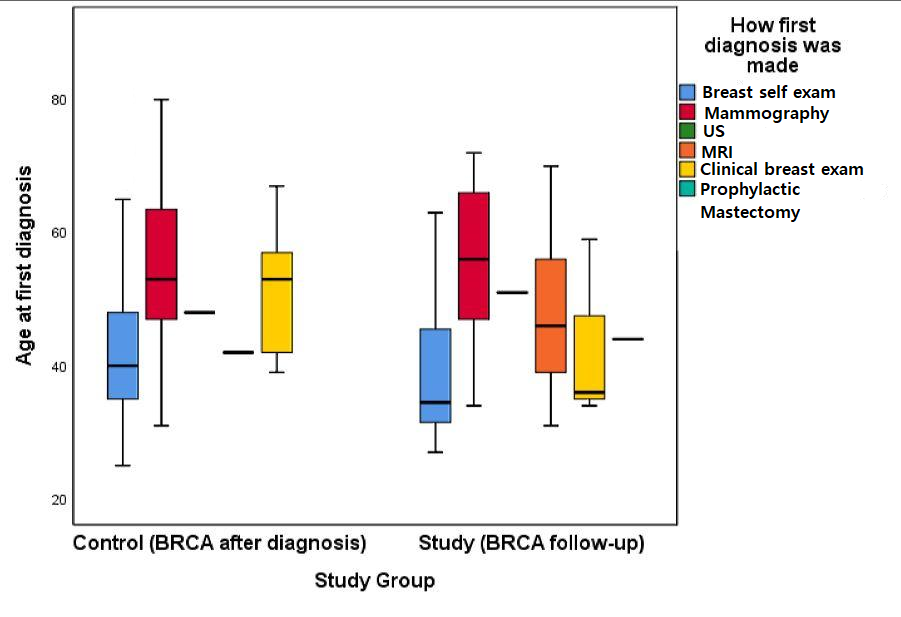

Supplement: Supplementary Figure 1 — How the first diagnosis was made with regard to age. BRCA-preDx awareness group (right) and BRCA-postDx awareness group (left). MRI, magnetic resonance imaging; US, ultrasound. [file Image_1.tif]

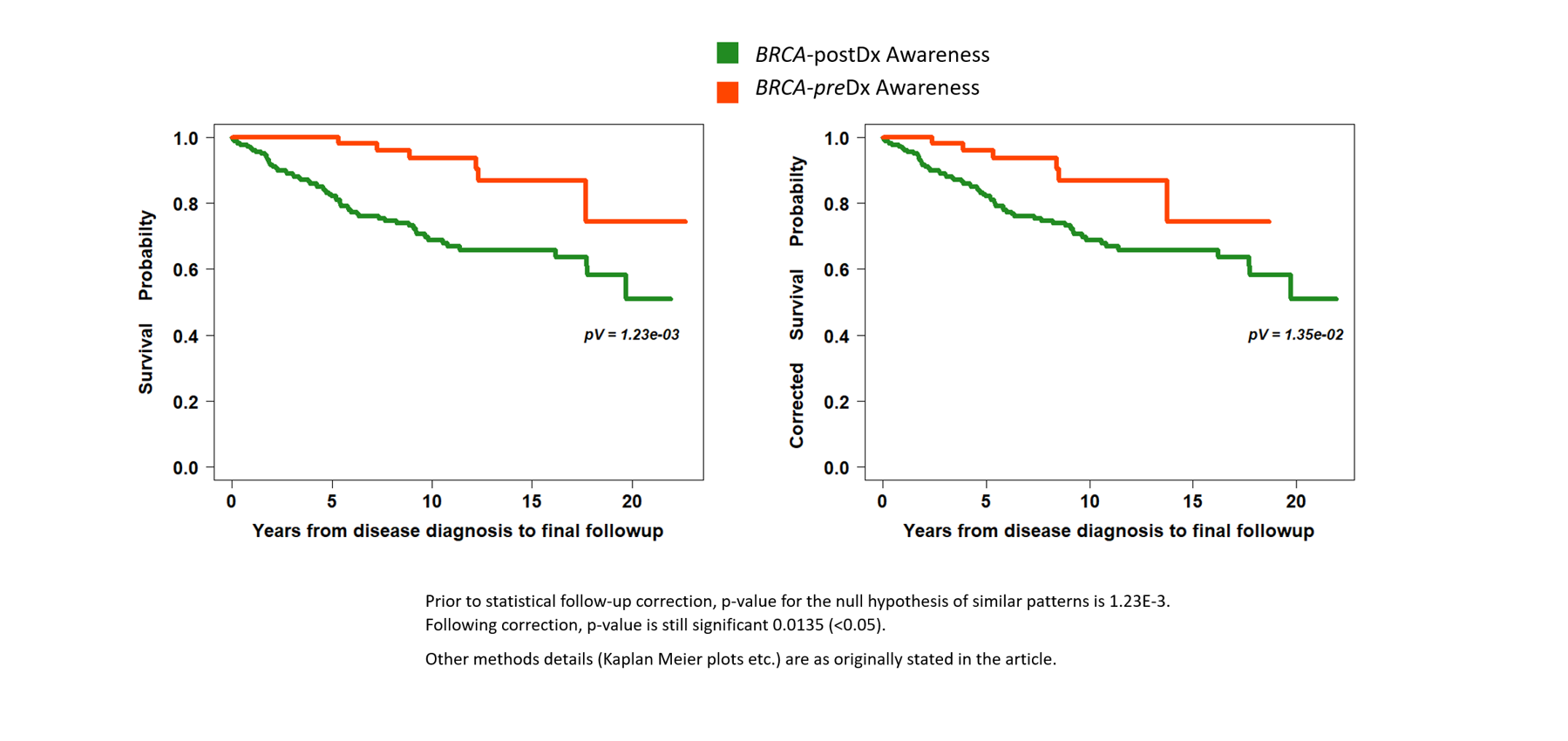

Supplement: Supplementary Figure 2 — Survival analysis following lead-time bias correction. BRCA-preDx awareness cohort and BRCA-postDx awareness cohort. [file Image_2.tiff]

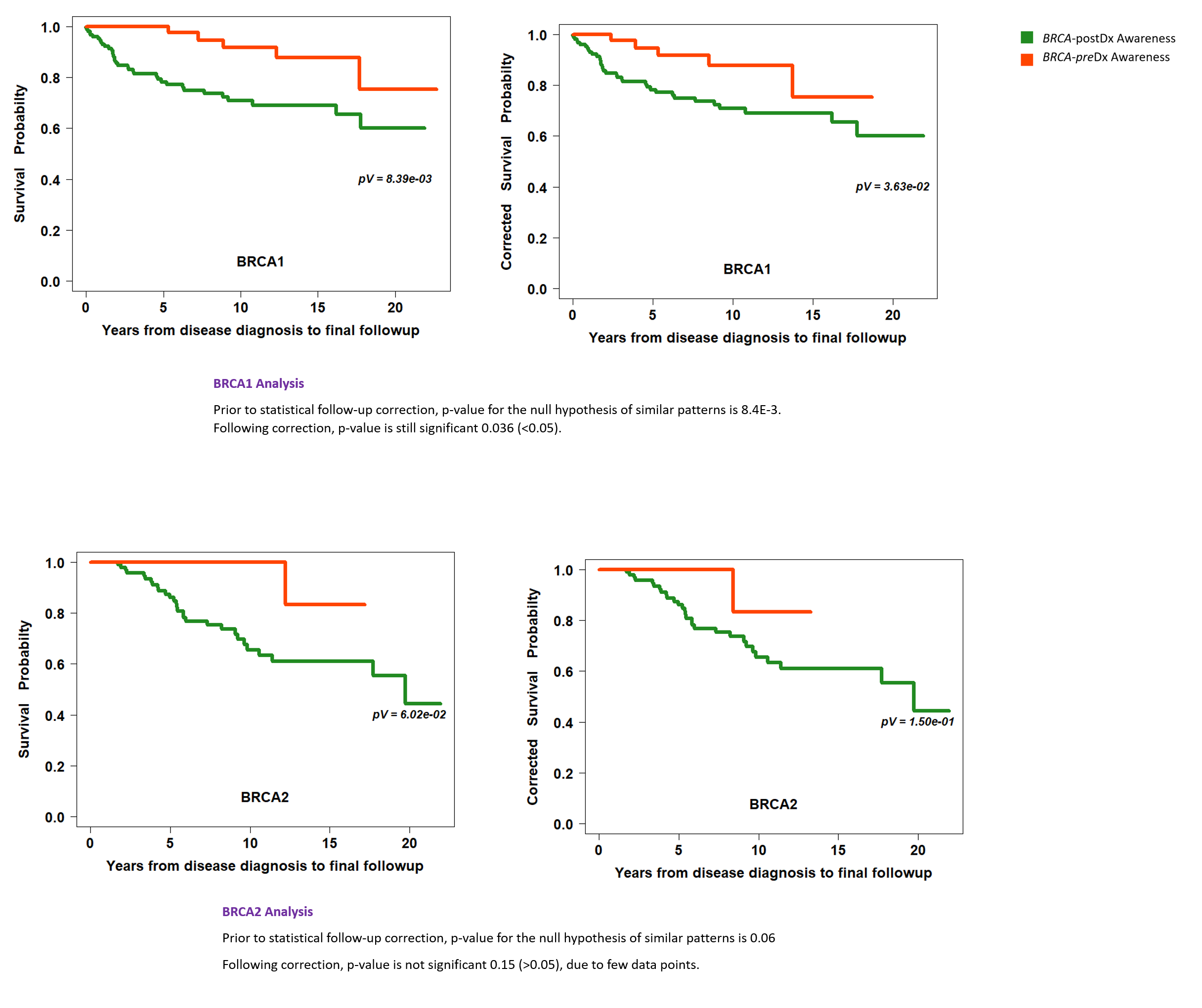

Supplement: Supplementary Figure 3 — Survival analysis according to BRCA1 and BRCA2 status. BRCA-preDx and BRCA-postDx awareness cohort separated according to BRCA1/2. [file Image_3.tif]
