# Supplementary material for: “High-Risk Breast Cancer Screening in BRCA1/2 Carriers Leads to Early Detection and Improved Survival After a Breast Cancer Diagnosis”
Source: Front Oncol. 2021 Sep 2;11:683656. doi: 10.3389/fonc.2021.683656 (PMC8443779; doi:10.3389/fonc.2021.683656)
Supplement: Supplementary Table 1 — Univariate and multivariate analyses of overall survival. [file Table_1.docx]

Supplementary Table 1

| **Variable** | **Overall survival** | | | | |
| --- | --- | --- | --- | --- | --- |
|  | **Univariate Analysis** | |  | **Multivariate Analysis** | |
|  | ***Hazard ratio (95% CI)*** | ***P value*** |  | ***Hazard ratio (95% CI)*** | ***P value*** |
| **Age at first diagnosis** | 0.983 (0.962-1.005) | 0.138 |  | 1.001 (0.966-1.038) | 0.935 |
| **Ancestry** |  | 0.963 |  |  | 0.985 |
| Ashkenazi Jewish | Reference | |  | Reference | |
| Sephardi Jewish | 0.935 (0.444-1.968) | 0.859 |  | 1.059 (0.329-3.408) | 0.923 |
| Other | 1.068 (0.283-4.036) | 0.922 |  | 0.958 (0.174-5.268) | 0.961 |
| **Family history** |  | 0.521 |  |  | 0.818 |
| First degree | Reference | |  | Reference | |
| Second degree  Or  Third degree | 0.716 (0.320-1.598) | 0.415 |  | 1.132 (0.382-3.354) | 0.823 |
| None | 0.954 (0.389-2.341) | 0.918 |  | 0.851 (0.239-3.032) | 0.803 |
| ***BRCA1* vs. *BRCA2*** | 1.185 (0.724-1.937) | 0.5 |  | 1.024 (0.452-2.322) | 0.954 |
| **ER** | 0.674 (0.408-1.111) | 0.122 |  | 0.516 (0.168- 1.579) | 0.246 |
| **PR** | 0.560 (0.342-0.916) | 0.021 |  | 3.179 (1.106- 9.135) | 0.032 |
| **HER2** |  | 0.199 |  |  | 0.638 |
| Negative | Reference | |  | Reference | |
| Positive | 2.331 (0.838-6.484) | 0.105 |  | 0.639 (0.247-1.653) | 0.355 |
| Not Applicable | 2.801 (0.901-8.704) | 0.075 |  | 1.031 (0.285-3.733) | 0.963 |
| **Grade** |  | 0.978 |  |  | 0.997 |
| Grade 1 | Reference | |  | Reference | |
| Grade 2 | 1.688 (0.380- 7.493) | 0.491 |  | 0.993 (0.201-4.919) | 0.993 |
| Grade 3 | 1.845 (0.444- 7.670) | 0.400 |  | 1.011 (0.204-5.010) | 0.989 |
| Low | 0 (0) | 0.989 |  | 1.344 (0) | 0.997 |
| Intermediate | 0 (0) | 0.978 |  | 1.124 (0) | 0.999 |
| High | 1.435 (0.130- 15.891) | 0.768 |  | 432.090 (0) | 0.934 |
| **T stage** |  | 0.003 |  |  | 0.887 |
| T1 | Reference | |  | Reference | |
| T2 | 1.682 (0.934-3.028) | 0.083 |  | 199.495 (0) | 0.943 |
| T3 OR T4 | 3.007 (1.543-5.861) | 0.001 |  | 279.514 (0) | 0.939 |
| Tis | 0.466 (0.108-2.007) | 0.306 |  | 284.170 (0) | 0.939 |
| **N stage** |  | <0.001 |  |  | 0.440 |
| N0 | Reference | |  | Reference | |
| N1 | 1.813 (0.963-3.414) | 0.065 |  | 1.723 (0.737-4.027) | 0.209 |
| N2 | 2.503 (1.280-4.895) | 0.007 |  | 1.365 (0.513-3.630) | 0.533 |
| N3 | 10.481 (4.935-22.259) | <0.001 |  | 3.120 (0.677-14.383) | 0.145 |
| **M stage** | 11.423 (6.250-20.878) | <0.001 |  | 7.536 (1.867- 30.425) | 0.005 |
| ***BRCA* postDx vs. *BRCA* preDx** | 0.341 (0.147-0.789) | 0.012 |  | 1.957 (0.576- 6.646) | 0.282 |
| **Surgery type** |  | <0.001 |  |  | 0.209 |
| Lumpectomy | Reference | |  | Reference | |
| Unilateral mastectomy | 1.546 (0.874-2.736) | 0.134 |  | 1.580 (0.702-3.556) | 0.269 |
| Bilateral mastectomy | 0.544 (0.247-1.199) | 0.131 |  | 0.743 (0.250-2.206) | 0.592 |
| Inoperable | 11.662 (5.328-25.528) | <0.001 |  | 3.671 (0.587-22.956) | 0.164 |
